# Ferritin in Adult-Onset Still's Disease: Just a Useful Innocent Bystander?

**DOI:** 10.1155/2012/298405

**Published:** 2012-03-25

**Authors:** Bella Mehta, Petros Efthimiou

**Affiliations:** ^1^Rheumatology Division, Lincoln Medical and Mental Health Center, New York, NY 10451, USA; ^2^Department of Medicine, Weill Cornell Medical College, New York, NY 10021, USA

## Abstract

*Background*. Adult-Onset Still's Disease (AOSD) is an immune-mediated systemic disease with quotidian-spiking fever, rash, and inflammatory arthritis. Hyperferritinemia is a prominent feature, often used for screening. *Methods*. The key terms “ferritin” and “hyperferritinemia” were used to search PubMed and Medline and were cross-referenced with “Still's Disease.” *Results*. Hyperferritinemia, although nonspecific, is particularly prevalent in AOSD. While most clinicians associate ferritin with iron metabolism, this is mostly true for the H isoform and not for the L isoform that tends to increase dramatically in hyperferritenemia. In these situations, hyperferritinemia is not associated with iron metabolism and may even mask an underlying iron deficiency. We review, in systematic fashion, the current basic science and clinical literature regarding the regulation of ferritin and its use in the diagnosis and management of AOSD. *Conclusion*. Serum hyperferritinemia in AOSD has been described for 2 decades, although its mechanism has not yet been completely elucidated. Regulation by proinflammatory cytokines such as interleukin (IL)-1b, IL-6, IL-18, MCSF, and INF-*α* provides a link to the disease pathogenesis and may explain rapid resolution of hyperferritinemia after targeted treatment and inhibition of key cytokines.

## 1. Introduction

Adult-Onset Still's disease (AOSD) is a rare, immune-mediated, multisystem inflammatory disorder characterized by quotidian spiking fevers, evanescent rash, and arthritis. It is frequently underdiagnosed and one of the main reasons for hospital admissions due to pyrexia of unknown origin (PUO).

The disease characteristically affects young individuals, with three quarters of the patients reporting disease onset between 16 and 35 years of age [1, 2]. Other symptoms include myalgia, inflammatory myopathy, liver abnormalities, pseudoangiocholitis, pleuritis, pericarditis, splenomegaly, pericardial tamponade and myocarditis, pulmonary fibrosis, pleural effusions, adult respiratory distress syndrome, interstitial nephritis, subacute glomerulitis, renal amyloidosis, collapsing glomerulopathy, thrombotic thrombocytopenic purpura, pure red cell aplasia, cranial nerve palsies, seizures, aseptic meningoencephalitis, and Miller-Fisher syndrome.

This syndrome was formerly thought to occur solely in children as systemic-onset juvenile idiopathic arthritis (SoJIA), previously known as juvenile Still's disease. Bywaters described in, 1971, a new disease entity that he named adult Still's disease; it involved adult patients who did not meet the criteria for classic rheumatoid arthritis (RA) but displayed features similar to those described in pediatric Still's disease [3].

Its etiology remains unknown. An infectious etiology has been postulated, although a definitive agent has never been identified and infectious agents are thought to be innate immunity triggers, leading to the clinical phenotype.

## 2. Methods

The key terms “ferritin” and “hyperferritinemia” were used to search Medline and Pubmed and cross-referenced with the key term “Still's disease” and “Adult-Onset Still's Disease” for all available full-text articles. Studies identified by the search strategies were assessed for relevance prior to inclusion in the paper. While the emphasis was on human studies, a few selected animal studies were included which provided important clues about the underlying pathophysiology.

## 3. Results

### 3.1. Regulation of Ferritin

A well-known feature of AOSD has increased levels of serum ferritin, usually five times, or more, above the upper limits of normal that at times may be extreme (>50,000 ug/dL). While by no means specific for the disease, serum hyperferritinemia is often used to aid the diagnosis of AOSD and serial serum levels are often used as a sort of biomarker to monitor response to treatment. Ferritin (apoferritin/iron-free ferritin) is a high-molecular-weight protein (450 to 600 kDa) composed of a nanocage of 24 assembled subunits. It can sequester up to 4500 iron atoms [24]. It is 8–12 nm in diameter which is as small as spherical viruses [25, 26]. It is found in many tissues and cell types. It is a necessary molecule for the cell's respiratory function where iron storage could cause free radical injury. The best-known function of ferritin is storage of iron. Ferritin captures the intracellular labile iron pool and thus “buffers” its effect. It is also an acute phase reactant, involved in inflammatory processes, which includes oxidative-stress-induced cell processes. Complementary DNA (antioxidant responsive element/Maf recognition element) along with mRNA (iron responsive element) regulates rate of ferritin synthesis [5, 27]. The cytoplasmic ferritin content is regulated by the translation of ferritin mRNAs in response to an intracellular pool of “chelatable” and “labile” iron. Inflammation is associated with increased production of ferritin by the histiocytomacrophage system and/or increased release from damaged hepatocytes. However, the precise mechanism and the regulation of this phenomenon are poorly defined [28]. Ferritin levels are increased in a few autoimmune diseases like RA but they hardly ever go as high as in AOSD [5].

### 3.2. Heme Oxygenase-1 Enzyme and Ferritin Expression

There has been a close association between the heme oxygenase-1 (HO-1) enzyme and ferritin expression in AOSD. HO-1 is an enzyme that degrades heme when induced to CO, Fe^2+^, and biliverdin. It is expressed by macrophages and endothelial cells in response to stress. Studies have shown that HO-1 mRNA increases in AOSD and that it may correlate with AOSD disease activity [15, 33], making it a potentially useful biomarker.

### 3.3. Ferritin Isoforms

**Figure 1:**
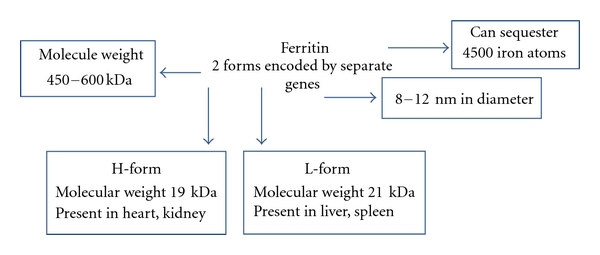


Isoelectric-focusing studies have identified several isoforms of ferritin. The acid form (H, heavy) is found chiefly in organs with low iron content, such as the heart and pancreas. In contrast, the base form (L, light) is found in organs (liver, spleen) and the histiocyte-macrophage system that has a significant iron storage capacity (Figure 1). The L-ferritin isoform is the one which is released in the circulation. The H-isoform has multiple catalytic sites and is faster than the L form. H-ferritin plays a major role rapid detoxification of iron and intracellular iron transport, whereas L-ferritin is involved in iron nucleation, mineralization, and long-term storage. The H : L ratio is normally constant in a cell, although it may change in hemochromatosis and other iron overload diseases [10–12]. The H : L ferritin ratio has not yet been defined in AOSD. In situations of iron overload, it may be advantageous to the cell to synthesize L-ferritin, since these ferritins are not only able to store higher iron amounts but can also retain iron more firmly and turn over iron more slowly than their H-ferritin counterparts [11]. In diseases like hyperferritinemia cataract syndrome, mutations in L ferritin have been documented [50]. However, no such study in AOSD has been contacted yet. A new isoform of ferritin has recently been described in breast cancer patients, HIV patients, and in pregnancy [51]. This finding suggests that there may be other isoforms that have not been identified yet and could explain the hyperferritinemia phenomenon in AOSD.

### 3.4. Ferritin and Disease Pathogenesis

The pathogenesis behind increased ferritin levels is thought to be cytokine mediated. Cytokines regulate ferritin synthesis at transcriptional, posttranscriptional, and translational stages. Cytokines implicated are IL1*α*, IL1*β*, IL18, tumor necrosis factor-*α* (TNF-*α*), interferon-*γ* (IFN-*γ*), macrophage-colony stimulating factor (M-CSF), IL6, and IL-18 [28, 52–55]. IL1*α*, IFN-*γ*, and TNF-*α* have shown to induce the expression of H-ferritin [54, 56, 57]. Translation of ferritin is induced by IL1*β*, IL-6, or TNF-*α* [58]. IL1*β* also affects ferritin regulation at a posttranscriptional stage [59]. The serum levels of Th1 cytokines and soluble IL-2 receptors are higher in AOSD than in other inflammatory joint diseases and have been correlated to the serum ferritin level [10].

 A study by Choi et al. on cytokines in AOSD showed significantly high IL-18, IFN-*γ*, and IL-8 levels in the sera of AOSD patients than healthy controls. Also, soluble IL-2 receptors level was increased only in active stage of AOSD which would indicate that soluble IL-2 receptor may be used as a potential marker for monitoring the disease activity in AOSD [42].

Cytokines may also affect ferritin translation indirectly by their ability to induce nitric oxide synthase (iNOS) and hence increase NO. NO in turn induces ferritin expression [53, 60].

The cytokine-mediated regulation suggests that inflammation can affect ferritin regulation.

There is also data to suggest that thyroid hormones play a role in ferritin expression [53, 61].

Lipopolysaccharide (LPS; endotoxin), an outer membrane component of several Gram-negative bacteria, elicits a variety of reactions that involve ferritin [53].

 In most studies, a threshold for serum ferritin levels of 1000 ng/mL, five times the upper limits of normal (40–200 ng/mL), has been used to suggest the presence of AOSD [28]. Very high levels ranging from 4000 ng/mL to 30,000 ng/mL are not uncommon, and even extreme levels as high as 250,000 ng/mL have been reported [2]. Ferritin levels in AOSD are usually higher than those found in patients with other autoimmune or inflammatory diseases [44]. It is not clear yet whether ferritin plays a role in the disease pathogenesis or it is just an acute phase reactant/silent bystander. In patients with chronic hepatitis C, ferritin and AST levels have been correlated, although increased ferritin does not seem to have a role in the extrahepatic manifestations of the disease. Also, in these patients, increased ferritin levels are not associated with the B-cell dysfunction represented by cryoglobulin and nonorgan-specific antibody production [18]. Additionally, there are several diseases associated with high ferritin levels that do not share any symptoms or signs of AOSD. The usefulness of serum ferritin is limited by the fact that elevated levels can also be seen in other diseases, such as infiltrative diseases (hemochromatosis, Gaucher's disease), infections (sepsis, HIV), malignancies (leukemia, lymphomas), and in the macrophage activation syndrome [62].  Table 1 illustrates all the diseases where ferritin levels increase or decrease, whereas Table 3 provides a summary of the studies of autoimmune diseases where ferritin is increased. Furthermore, there are several well-documented reports of AOSD without increase in ferritin levels, hinting on possible different underlying mechanisms [37].

Interestingly, serum ferritin levels often correlate with disease activity and can normalize when the disease goes into remission [47, 49, 63]. Ferritin is known to release free Fe^2+^ ions, which catalyze the reaction leading to the formation of free OH^−1^ radicals, although it can also chelate these free Fe^2+^ ions, thereby limiting the deleterious effects of oxidative stress [64, 65]. The unresolved question is whether ferritin acts as a buffer to minimize the pathogenic effects of free radicals or is it the one to cause the release of them.

### 3.5. Ferritin Glycosylation in AOSD

In healthy individuals, 50–80% of ferritin is glycosylated and the attachment of glucose molecules at the surface of the ferritin molecule may provide protection against proteolytic enzymes. There have been several studies which point to the fact that AOSD patients have low glycosylation levels (<20%) [37, 40]. Abnormally, low levels of ferritin glycosylation were shown to be a more specific, albeit less sensitive, diagnostic test for AOSD. Unfortunately, this test is not readily available in clinical practice, hence limiting its usefulness. Moreover, ferritin glucosylation remains low both during active state and in remission, unlike serum ferritin levels [40]. The pathogenic mechanisms underlying the decrease in glycosylation are poorly defined. A probably theory could be that, due to excess of ferritin, the glycosylation process could be saturated. In addition to saturation of glycosylation mechanisms, abnormalities that are more specific of AOSD have been suggested, particularly decreased clearance of nonglycosylated proteins by the histiocyte-macrophage system.

The defect in ferritin glycosylation, although more specific for the diagnosis of AOSD than serum ferritin, is by no means pathognomonic for the disease and has several limitations. Individual patients can have normal levels of glycosylation and low glycosylation levels can be seen in other inflammatory disorders and in a few patients with infectious diseases [37]. Glycosylated ferritin cannot be used to monitor disease activity or response to treatment, as it remains low for many months after the disease goes into remission [40]. Glycosylated ferritin (<20%) has a sensitivity of 78% and specificity of 64%. When glycosylated ferritin levels are combined with a fivefold serum rise in ferritin, the sensitivity fell to 43% and specificity rose to 93% [37]. Therefore, the combined use of both parameters has been suggested and included in the Fautrel et al. criteria.

### 3.6. Ferritin Association with Atherosclerosis

AOSD is one of the diseases under the banner of autoinflammatory diseases, a new disease category where atherosclerosis has been suggested as a possible member. Ferritin has also been implicated in the pathogenesis a number of diseases (Table 2). It has been described more clearly and significantly in atherosclerosis [17, 29, 66–68]. Epidemiological studies have linked elevated serum ferritin levels with an increased risk for coronary artery disease (CAD) and myocardial infarction (MI) [63]. This finding led to the “iron hypothesis” which suggested a link between abnormal iron storage and atherosclerosis. Furthermore, the hemochromatosis gene (HFE), C282Y, has been associated with an increased risk of CAD and cardiovascular mortality [69, 70]. There is an ongoing debate whether ferritin acts as a prooxidant, releasing free iron that was previously bound to it, or antioxidant, sequestering excess unbound iron. Excessive iron in tissues can catalyze the formation of oxygen-free radicals that can lead to low-density lipoprotein (LDL) oxidation, a trigger for the development of atherosclerosis.

### 3.7. Mutated Ferritin Theory

During infection or inflammation, iron is sequestered in the ferritin contained inside macrophages, and, as a result, serum iron decreases. This artificial “iron deficiency,” which in reality is scarcity in the midst of plenty, is thought to be protective for the host, depriving invading microorganisms from much needed iron [71]. Some research suggested that iron release is defective due to the hyperferritinemia in AOSD [72, 73]. Reports of iron supplementation successfully treating systemic-onset juvenile chronic arthritis [74] prompted the performance of iron studies on AOSD patients, showing iron deficiency, and suggested that low-dose intravenous iron supplementation could be effective in AOSD patients with anemia [48, 74, 75]. The investigators suggested that intravenous iron could by-pass macrophage trapping and become directly available for erythropoiesis. This strategy could prove to be effective in anemic AOSD patients who often have normal or increased iron stores. Despite the massive amounts of circulating ferritin, its saturation with iron molecules since AOSD is not associated with iron overload [40, 76, 77]. This has also been proven with the use of automated analyzers that measure the transferring receptors in the serum [78]. Moreover, since the serum-transferring receptor concentration is not altered in inflammatory states, it may be a more useful test than serum ferritin in assessing the iron stores in AOSD [79]. The defective release of iron from ferritin could be secondary to the presence of a mutant form of ferritin, which could also explain the defect in ferritin glucosylation seen in AOSD.

## 4. Conclusion

Very high and often extreme serum ferritin levels have been described in AOSD for more than 2 decades now. While widely thought to be an acute phase reactant, ferritin could be intimately involved in the disease pathogenesis as an oxygen radical donor or scavenger or via a yet to be defined mechanism, possibly including mutated ferritin. Further research is warranted to bridge the knowledge gap and identify the missing links.

## Figures and Tables

**Table 1:** Diseases in which ferritin levels increase or decrease.

Ferritin levels increase	Ferritin levels decrease
Adult-Onset Still's Disease [2]	Anemia [4]
Rheumatoid arthritis [5]	Restless leg syndrome [6]
Macrophage activation syndrome [7]	Neuroferritinopathy [8]
Multiple sclerosis [9]	Vitamin C deficiency
Haemochromatosis [10–12]	Celiac disease [13]
Haemosiderosis [10–12]	Hypothyroidism [14]
Haemophagocytic lymphohistiocytosis [15, 16]	
Diabetes [17]	
Hepatitis C infection [17, 18]	
Glomerular diseases [19]	
Hyperferritinemia cataract syndrome [20]	
Chronic blood transfusions [21]	
Non-HIV infections [22]	
Malignancies [22]	
Type 1 Gaucher's disease [23]	

**Table 2:** Ferritin implicated in the pathogenesis of the following diseases.

Atherosclerosis [29]
Diabetes [17]
Parkinson's disease [30]
Alzheimer disease [31]
Pulmonary disease [32]

**Table 3:** Hyperferritinemia in Adult-Onset Still's Disease patient cohorts (*n* ≥ 4).

Name	Year	Number of patients	Results
Zandman-Goddard and Shoenfeld [34] (Israel)	2008	403 autoimmune disease patients	Hyperferritinemia in 23% SLE patients, 15% dermatomyositis, 8% multiple sclerosis, 4% rheumatoid arthritis
da Costa et al. [35] (Brazil)	2011	150 multiple sclerosis patients	8% of MS patients had hyperferritinemia
Lian et al. [36] (China)	2010	48 AOSD patients and 86 non-AOSD patients	Significantly higher levels of hyperferritenemia in AOSD
Fautrel et al. [37] (France)	2001	49 AOSD and 120 control group patients	Mean ferritin level was significantly higher in AOSD than in control group
Sobieska et al. [38] (Poland, Germany, Switzerland, France)	1998	27 AOSD and 10 pediatric Still's Disease patients.	Mean ferritin level was significantly higher in AOSD than children
Schiller et al. [39] (Austria)	1998	4 AOSD patients	All ferritin levels >5000 ng/mL
Vignes et al. [40] (France)	2000	14 AOSD patients	Mean ferritin level was 6350 ng/mL
Uppal et al. [41] (Kuwait)	2007	28 AOSD patients	Hyperferritinemia in 89% patients
Choi et al. [42] (Korea)	2003	17 AOSD patients	Hyperferritinemia in 14 patients
Arlet et al. [43] (France)	2006	6 AOSD patients with haemophagocytic syndrome	Serum ferritin level above 10,000 ng/mL in 5 patients
Coffernils et al. [44] (Belgium)	1992	10 AOSD patients	Hyperferritinemia in 8 patients
Ota et al. [45] (Japan)	1987	5 AOSD patients, 7 RA patients	Mean ferritin levels in AOSD were 21,565 ng/mL, whereas, in RA, mean levels were 181 ng/mL
Baxevanos et al. [46] (Greece)	2011	22 AOSD patients	Hyperferritinemia in 21 patients
Akritidis et al. [47] (Greece)	1997	9 AOSD patients	8 patients had ferritin levels above 4000 ng/mL
Montecucco et al. [48] (Italy)	1995	4 AOSD patients, 7 RA patients	All 4 AOSD patients had hyperferritinemia and had mean ferritin greater than RA patients
Van Reeth et al. [49] (France)	1994	20 AOSD patients	Ferritin levels are higher in active AOSD than in inactive AOSD
