# Erratum to “Ferritin in Adult-Onset Still's Disease: Just a Useful Innocent Bystander?”

**DOI:** 10.1155/2012/254858

**Published:** 2012-08-26

**Authors:** Bella Mehta, Petros Efthimiou

**Affiliations:** ^1^Rheumatology Division, Lincoln Medical and Mental Health Center, New York, NY 10451, USA; ^2^Department of Medicine, Weill Cornell Medical College, New York, NY 10021, USA

There is an error in the citations of our previous paper as reference [51] G. Zandman-Goddard, M. Blank, P. Langevitz et al., *“Antiserum amyloid component P antibodies in patients with systemic lupus erythematosus correlate with disease activity,”* Annals of the Rheumatic Diseases, vol. 64, no. 12, pp. 1698–1702, 2005 should be replaced by the following references: [1] Li, X., et al., “*Epidermal Growth Factor-Ferritin H-Chain Protein Nanoparticles for Tumor Active Targeting,*” Small, 2012; [2] Jezequel, P., et al., “*Validation of tumor-associated macrophage ferritin light chain as a prognostic biomarker in node-negative breast cancer tumors: A multicentric 2004 national PHRC study,*” International Journal of Cancer, 2012. 131(2): p. 426–37. [3] Pitcher, J., et al., “*Disruption of neuronal CXCR4 function by opioids: preliminary evidence of ferritin heavy chain as a potential etiological agent in neuroAIDS,*” Journal of Neuroimmunology, 2010. 224(1-2): p. 66–71. [4] Rosen, H. R., “*Placental isoferritin-associated p43 in pregnancy and breast cancer: minireview,*” Neoplasma, 1996. 43(6): p. 357–62.

